# Expression of Concern on “Seminal miRNA Relationship with Apoptotic Markers and Oxidative Stress in Infertile Men with Varicocele”

**DOI:** 10.1155/bmri/9820717

**Published:** 2026-07-28

**Authors:** BioMed Research International

EXPRESSION OF CONCERN: T. Mostafa, L. A. Rashed, N. I. Nabil, I. Osman, R. Mostafa, and M. Farag, “Seminal miRNA Relationship with Apoptotic Markers and Oxidative Stress in Infertile Men with Varicocele,” *BioMed Research International* 2016, no. 1 (2016): 4302754, https://doi.org/10.1155/2016/4302754.

This Expression of Concern is for the above article, published online on 26 December 2016 in Wiley Online Library (http://wileyonlinelibrary.com), and has been agreed to by the Editor‐in‐Chief, Yoel Klug; and John Wiley & Sons Ltd. The Expression of Concern has been agreed due to figure concerns identified with Figures 2, 3 and 5, which were raised by a third party. The concerns are summarized as follows: In Figures 2a, 2b, 2e, 3a, 3b, 3e and 5a, there are groups of 3 datapoints which appear to be repeated at multiple points in the graphs. The journal contacted the authors and requested the original data and an explanation. However, the authors were unable to provide the detailed raw data due to the passing of their statistician since the original publication. Therefore, the journal has decided to issue an Expression of Concern to inform and alert readers.

